# Investigating social orienting in children with Phelan-McDermid syndrome and ‘idiopathic’ autism

**DOI:** 10.1186/s11689-024-09564-7

**Published:** 2024-11-19

**Authors:** Antonia San José Cáceres, Emma Wilkinson, Jennifer Cooke, Victoria Baskett, Charlotte Blackmore, Daisy Victoria Crawley, Allison Durkin, Danielle Halpern, María Núñez, Page Siper, Declan G. Murphy, Jennifer Foss-Feig, Alexander Kolevzon, Eva Loth

**Affiliations:** 1 Department of Forensic and Neurodevelopmental Sciences, Institute of Psychiatry, Psychology & Neuroscience, King’s College London, De Crespigny Park, London, UK; 2 Departamento de Psiquiatría del Niño y del Adolescente, Instituto de Investigación Sanitaria Gregorio Marañón, Hospital General Universitario Gregorio Marañón, Madrid, Spain; 3 Centro Investigación Biomédica en Red Salud Mental, CIBERSAM, Instituto Carlos III, Madrid, Spain; 4 Seaver Autism Center for Research and Treatment, Department of Psychiatry, Icahn School of Medicine at Mount Sinai, New York, NY USA; 5 Department of Psychology, University at Albany State University of New York, NY, USA; 6 Department of Clinical, Educational, and Health Psychology, University College London, London, UK; 7 Department of Pediatrics, Icahn School of Medicine at Mount Sinai, New York, NY USA; 8 Departamento de Psicología Básica, Universidad Autónoma de Madrid, Madrid, Spain; 9 Sackler Institute for Translational Neurodevelopment, Institute of Psychiatry, Psychology & Neuroscience, King’s College London, De Crespigny Park, London, UK

**Keywords:** PMS, Phelan-McDermid syndrome, Idiopathic autism, Auditory social orienting

## Abstract

**Background:**

Phelan-McDermid syndrome (PMS) is a rare genetic syndrome characterized by developmental delay/intellectual disability, absent or delayed speech, physical dysmorphic features and high rates of autistic features. However, it is currently unknown whether people with PMS have similar neurocognitive atypicalities to those previously identified in idiopathic autism. Disruption in social orienting has previously been suggested as an early hallmark feature of idiopathic autism that impacts social learning and social interaction.

**Methods:**

This study used a semi-naturalistic task to explore orienting to social versus non-social stimuli and its relation to clinical features in individuals diagnosed with PMS, autism, and neurotypical children recruited in the United States and the United Kingdom.

**Results:**

At the group level, autistic and neurotypical children responded on average more often to social than non-social stimuli, while children with PMS responded similarly to both stimulus types. Both clinical groups responded significantly less often to social stimuli than neurotypical children. In addition, we found considerable variability in orienting responses within each group that were of clinical relevance. In the autism group, non-social orienting was associated with mental age, while in the PMS group social and non-social orienting were related to strength of autistic features.

**Conclusions:**

These findings do not support specific social motivation difficulties in either clinical group. Instead, they highlight the importance of exploring individual differences in orienting responses in Phelan-McDermid Syndrome in relation to autistic features.

**Trial registration:**

NA.

**Supplementary Information:**

The online version contains supplementary material available at 10.1186/s11689-024-09564-7.

## Background

Phelan-McDermid syndrome (PMS), also known as 22q13.3 deletion syndrome, is a rare neurodevelopmental condition caused by a sequence variant in the *SHANK3* gene or deletion on the long arm of terminal chromosome 22 (OMIM 606230; Phelan & McDermid, [1]; Phelan, [2]). PMS is characterized by a heterogeneous array of clinical features, including hypotonia, absent or delayed speech, and in the vast majority of individuals (96%), intellectual disability [2, 3]. Furthermore, approximately 65% of individuals with PMS are diagnosed with autism spectrum disorder (henceforth “autism”^1^) (4). Yet, the neurocognitive profile of PMS is not well understood and the extent to which individuals with PMS have similar characteristics to “idiopathic” autistic individuals (i-autism) remains unknown. Although the clinical profile in PMS may be expected to be more homogeneous than that of i-autism, reports attest to a considerable amount of clinical heterogeneity [4–6].

Atypical attention patterns are consistently found in autism [7, 8], with some theories emphasizing either broad attention differences or differential attention to specifically social information [9, 10]. The social motivation hypothesis of autism proposes that reduced spontaneous attention to social information is a primary feature that affects the development of social cognition and social skills (e.g., [10–12]. Diminished *spontaneous* orienting to social stimuli (e.g., a smile), but not non-social stimuli (e.g., objects), is thought to be one of the earliest manifestations of reduced social motivation. Eye-tracking studies in infants with increased familial likelihood for autism have demonstrated decreased spontaneous attention to social information during the viewing of static images and dynamic social scenes [13, 14]. Failure to respond to one’s own name being called is also identified as among one of the earliest signs of autism [15, 16]. A behavioral paradigm involving the presentation of social (e.g., clapping, humming) and non-social (e.g., telephone, car horn) sounds revealed that autistic preschool-age children showed on average diminished orienting to social sounds, relative to both typically developing children and children with developmental delay of comparable mental age [17]. Taken together, these data support the notion of an early social attention difficulty in autism.

Social attention difficulties have also been linked to social-communication features in several studies [18–21]. For example, Murias et al. [18] found that in autistic toddlers, less attention to social bids was associated with lower scores on several measures of socio-communicative behavior. Relatedly, autistic toddlers who looked less often at social scenes showed more autistic core features [19]. Studies with autistic adolescents and adults indicate that this relationship between social attention and social-communication features persists through to adulthood [11, 22, 23].

A separate hypothesis proposes that autism may involve more global, domain-general attentional differences, resulting in difficulties orienting and shifting of attention regardless of stimulus type [24, 25]. Supporting this idea, one study found that autistic children had difficulty with attention disengagement as compared to typically developing controls, regardless of the social nature of the stimuli [26]. Sasson et al. [27] also found that across non-social and social arrays, autistic children showed domain-general difficulties in disengagement. There is also evidence that atypical neurobiological development of attention networks in autism contributes to global impairments in attention [28]. Given the considerable clinical and etiological heterogeneity in autism, it is plausible that some individuals have domain-general attentional difficulties, others attend less to specifically social information, and that attention patterns vary between individuals.

In contrast to the relatively robust literature on social and non-social attention in autism, there has been little research examining attentional patterns in individuals with PMS. To address this gap in knowledge, the present study pooled data from two clinical natural history studies of individuals with PMS carried out simultaneously in the United States (US) and the United Kingdom (UK). Both sites employed an established social orienting paradigm [17] to investigate whether individuals with PMS show less orienting to specifically social stimuli or present with general difficulties in attention orienting. Our aims were to examine: (1) average group differences between the PMS, i-autism and neurotypical groups in orienting patterns, and (2) the relationships between individual differences in orienting responses and measures of social-communicative features, cognitive and social adaptive functioning.

The social motivation hypothesis predicts that individual with PMS, like autistic children, would orient specifically less to social but not non-social stimuli, and that the frequency of social orienting would be negatively related to autistic features and positively related to social adaptive functioning skills in both groups. Alternatively, if PMS-related attention difficulties were more linked to their overall intellectual level, we would expect to find equal impairments for both social and non-social orienting, with these generalized impairments relating to intellectual functioning. Studying these differences will bring us closer to understanding the cognitive profile of PMS individuals, to ultimately provide them with better and more tailored care.

## Methods

### Participants

The study was conducted simultaneously at two separate sites: the Seaver Center for Research and Treatment at Mount Sinai (New York) in the US, and the Institute of Psychiatry, Psychology and Neuroscience (London) in the UK. Approval was obtained by the respective ethics committee at each site. Children and young adults with PMS were recruited through the PMS family foundations in the US or in the UK. Additionally, two British families were recruited via referral from genetic clinics in the local area. In total, the study included 67 participants with PMS: 46 from the US and 21 from the UK. PMS diagnosis was confirmed through molecular genetic testing. Fifty-four participants autistic participants also participated: 23 from the research center in the US, and 31 from special needs schools in the wider London area (UK). All autistic participants were previously diagnosed using the Diagnostic and Statistical Manual for Mental Disorders, Fourth Edition Revised or Fifth Edition (DMS-IV-TR or DSM-5; American Psychological Association, [29] and [30] respectively), or the International Classification of Diseases (ICD-10; World Health Organization, [31]) criteria. Participants ranged in age from 1.5 to 24 years of age (or 19 to 293 months). Across sites, greater numbers of males were included in the i-autism groups, whereas sex was distributed evenly in the PMS group, consistent with published reports. To match the delayed language and cognitive profile of the PMS group, only minimally verbal (no words, single words, or one-clause sentences) and/or intellectually disabled autistic individuals were enrolled (Table 1) (intellectual disability encompasses individuals with IQ > = 70, and/or impaired adaptive functioning and these difficulties are apparent during development). For referential purposes to compare responses to a non-clinical sample, a group of 28 neurotypical/typically developing (TD) children were also recruited in the UK following the same procedures as with the PMS and i-autism individuals. TD children ranged in age from 1.5 to 6 years.

### Procedure

#### Autistic features

To assess autistic features, participants in the PMS and i-autism groups were administered the Autism Diagnostic Observation Schedule 2nd Edition (ADOS-2; [32]) and caregivers were administered the Autism Diagnostic Interview – Revised (ADI-R; [33]). The Calibrated Severity Score (CSS; calculated from [34] and [35]), the Total score, and the Social Affect and Repetitive and Restrictive Behaviors subscores from the ADOS-2, as well as the Language/Communication, Reciprocal Social Interactions, and Repetitive Behaviors/Interests domain scores from the ADI-R were used to characterize clinical severity. Although this is not confirmatory of diagnosis, across sites, 66 of 67 individuals with PMS (99%) had an ADOS-2 performed and 64 (97%) met criteria for autism or autism spectrum. Similarly, for the ADI-R, 66 of 67 PMS participants had the instrument administered and 58 (89%) met criteria for abnormalities in reciprocal social interaction (domain A), 58 (89%) for abnormalities in communication (domain B), and 45 (68%) for restricted, repetitive, and stereotyped patterns of behavior (domain C). 83% of individuals with PMS at the US site also met criteria for autism based on consensus diagnosis using ICD or DSM criteria and informed by the ADOS-2 and ADI-R. All autistic participants met clinical ICD or DSM criteria for autism.

#### Cognitive profile

In all groups, mental age was assessed using a range of measures depending on the participant’s language and ability level. For example, the Mullen Scales of Early Learning (MSEL; [36]) was used for children below age five years and/or with mental age estimates below the floor of other age-appropriate test options. For those above this cut off, the US site used the Stanford Binet Intelligence Scales – Fifth Edition [37], the Differential Ability Scales II - Early Years [38], or the Leiter International Performance Scale [39]. The UK site used the Wechsler Abbreviated Scales of Intelligence - 2nd Edition [40] or a combination of the Raven’s Coloured Progressive Matrices [41] and the British Picture Vocabulary Scale 3rd Edition [42]. Verbal mental age (VMA) was computed from the average of the Receptive Language and the Expressive Language mental ages of the MSEL or calculated from the verbal intelligence quotient (VIQ) subscales of other instruments (i.e., VDQ = VMA/CA*100). Similarly, non-verbal mental age (NVMA) was calculated using the average of the Visual Reception and Fine Motor mental ages of the MSEL or non-verbal IQ of the other measures.

The Vineland Adaptive Behavior Scales 2nd Edition (Vineland-II; [43]) Survey Interview Form was administered to parents to assess their child’s adaptive functioning in everyday life. Standard scores (mean = 100, SD = 15) from three main domains (Communication, Socialization, and Daily Living Skills), as well as the Adaptive Behavior Composite (ABC) were used to relate social orienting variables to everyday functioning.

Table 1 presents the chronological and mental ages, Vineland-II, ADOS-2, and ADI-R scores for each group. Despite significant differences between groups in developmental level and most of the adaptive behavior domains, the i-autism and PMS groups did not significantly differ, in social communication features on the ADOS-2, ADI-R, or the Vineland-II. However, in terms of restrictive and repetitive behaviors, the i-autism group scored on average significantly higher than the PMS group on the ADI-R.

**Table 1:** Descriptors for age and sex and social variables by diagnosis (Mean (SD) [range])

	PMS	i-autism	TD	*p* ^a^	Post-hoc
*N*	67	50	28		
Sex (m: f)	33:34	46:4	17:11	< .001	PMS – autism** PMS - TD ns autism - TD**
CA (months)	92.43 (51.96) [19–293]	90.77 (42.07) [34–227]	48.73 (19.71) [19–83]	< .001	PMS - autism ns PMS > TD*** autism > TD***
NVMA (months)	22.27 (21.95) [4.95–89.01]	36.67 (38.14) [1-246.03]	60.71 (37.12) [17–143]	< .001	PMS < autism*** PMS < TD*** autism < TD***
VMA (months)	23.35 (22.41) [2.0-86.94]	33.04 (22.79) [4.5-132.13]	64.60 (44.97) [11–179]	< .001	PMS < autism*** PMS < TD*** autism < TD***
Vineland Communication	50.40 (14.86) [21–91]	62.09 (19.06) [36–116]	111.21(14.54) [84–141]	< .001	PMS < autism*** PMS < TD*** autism < TD***
Vineland Daily Living	51.52 (13.91) [21–82]	61.72 (15.48) [34–97]	98.57 (12.92) [71–125]	< .001	PMS < autism** PMS < TD*** autism < TD***
Vineland Socialization	57.67 (15.10) [20–101]	60.02 (15.6) [40–108]	101.36 (11.44) [79–126]	< .001	PMS –autism ns PMS < TD*** autism < TD***
Vineland Composite	51.61 (13.11) [20–83]	60.70 (13.81) [39–92]	102.86 (13.47) [84–131]	< .001	PMS < autism** PMS < TD*** autism < TD***
ADOS CSS	7.00 (2.04) [1–10]	7.04 (1.78) [3–10]	NA	.905	NA
ADOS Total	17.39 (5.83) [2–27]	17.62 (5.37) [6–28]	NA	.962	NA
ADOS SA	14.21 (4.79) [2–20]	13.58 (4.24) [3–20]	NA	.280	NA
ADOS RRB	3.18 (2.08) [0–8]	4.04 (2.21) [0–8]	NA	.051	NA
ADI-R RecSoc	18.86 (8.98) [0–30]	20.69 (8.00) [0–30]	NA	.454	NA
ADI-R Communication	12.54 (5.54) [0–25]	14.62 (5.93) [0–23]	NA	.075	NA
ADI-R RRB	3.47 (2.37) [0–10]	6.69 (1.89) [3–10]	NA	< .001	PMS < autism*

a = p for Kruskall-Wallis or Mann-Whitney depending on the number of groups to compare; ns = non-significant; * p < .05; ** p < .01; *** p < .001; *NA = not applicable;* CA = Chronological Age; NVMA = NonVerbal Mental Age; VMA = Verbal Mental Age; Vineland = Vineland Adaptive Behavior Scale, ADOS = Autism Diagnostic Observation Schedule; CSS = Calibrated Severity Score; SA = SocioAffective; RRB = Repetitive and Restrictive Behaviors; ADI-R = Autism Diagnostic Interview - Revised; RecSoc = Reciprocal Social Interaction.

#### Social orienting task

To assess social attention, we used the established Dawson et al. [17] paradigm. Social orienting was always completed before the ADOS-2 at the US site and afterwards at the UK site. For this task, one examiner sat in front of the child while engaged in a neutral activity, such as looking at a picture book. A second examiner waited until the child was engaged in the activity, and then delivered each stimulus. The auditory stimuli consisted of four social (humming a neutral tone; calling the child’s name; snapping fingers; patting hands on thighs) and four non-social (timer beep; phone ringing; whistle; car horn) sounds. Each stimulus was delivered three times per trial with a one second interval in between. For each stimulus, if the child turned his/her head and/or eyes toward the stimulus within 15 s of delivery, it was coded as successful orienting (i.e., 1). If s/he did not turn his/her head and/or eyes toward the stimulus, or took longer than 15 s, non-orienting was coded (i.e., 0). The total number of orienting responses was computed separately for social and non-social stimuli. Therefore, the possible scores for responses to either social or non-social stimuli could range between 0 and 4, with higher scores indicating better orienting responses. To explore the general orienting tendency to sounds, a total composite score was also calculated by adding all social and non-social positive responses. All sessions were videotaped and reviewed by a third rater to resolve any coding discrepancy between examiners.

Of note, small differences in administration existed across sites. At the US site, all stimuli were administered from around the room (behind-left, behind-right, front-left, front-right), with order and location of the stimuli counterbalanced across participants (*n* = 8 total trials). At the UK site, all eight sounds were presented following a pseudo-random stimuli presentation sequence, from the front-left and front-right, and then again from the behind-left and behind-right, or vice versa in a counterbalanced fashion (*n* = 16 total trials). For the UK site, post-testing analyses revealed significantly more orienting responses when stimuli were delivered from the front rather than from the back for PMS and autistic individuals (all *p* < .016 see Appendix 1a) and not for TD children (all *p* > .55), but first or second position order (i.e. front then back versus back then front) made no difference, indicating a lack of priming effect (all *p* > .060 – see Appendix 1b). Therefore, to account for the double presentation of stimuli and to be equivalent to the results from the US, the total number of orienting responses were divided by two. Three UK participants were too agitated and could not be administered all the stimuli; in those cases, the response to their total number of stimuli presented (if at least two bids per condition were administered) was pro-rated by using the average score of the valid trials.

#### Analysis

All analyses were completed using IBM SPSS^®^ version 26, and figures were created using the *ggplot2* library [44] in *R*. Group comparisons between all three groups were conducted using Kruskal-Wallis tests. Since responses on the orienting conditions were nonnormally distributed, the Wald test was used to explore condition by diagnosis effects. Mann-Whitney U tests were used in all cases for posthoc tests. To explore the weight of the independent variables of interest (i.e. verbal and non-verbal mental ages, ADOS Calibrated Severity Scores and Vineland Socialization sub-domain scores) these were entered into a linear regression model with social or non-social orienting responses as dependent variable, divided by group.

## Results

### Social orienting results

**Fig. 1:**
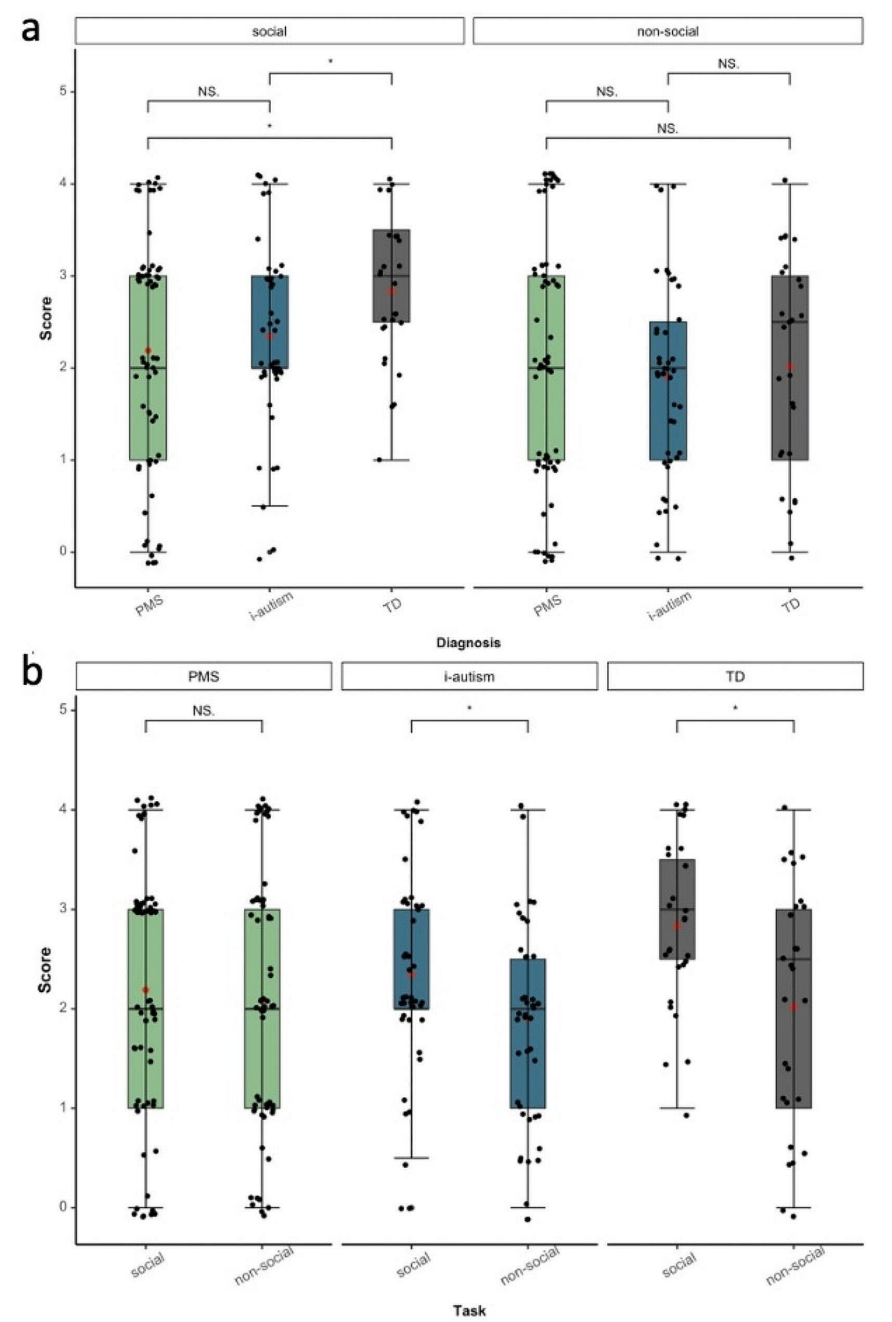
Distribution of scores and comparison **(a)** by type of stimuli between groups, and **(b)** within groups by type of stimuli. Bars between box plots represent significant values (NS. = non-significant; * *p* < .05) for mean differences between groups. Red dots indicate the group mean

Mean-group differences were explored in relation to the number of orienting responses. Figure 1 shows the distribution of scores for stimulus type (i.e., social vs. non-social) by group. There was a significant effect of group (*W*_(2)_ = 2.61, *p* < .001), a significant effect of condition (*W*_(1)_ = 16.27, *p* < .001) and a significant group by interaction effect (*W*_(2)_ = 5.83, *p* < .001). Post-hoc analyses showed that in the social condition, the i-autism and PMS groups scored on average significantly lower than TD children while the groups did not significantly differ from one another in the non-social condition. Within-group comparisons revealed that the PMS group responded on average to social and non-social stimuli at a similar rate, while the i-autism group showed a similar pattern to the TD group by orienting, on average, more often to social than non-social stimuli.

However, as can also be seen from individual scores overlaid onto the box plots, there was a wide variability in responses in all groups. Except for TD children in the social condition, scores covered the full range of scores from 0 to 4 in each condition.

To check whether a clinical diagnosis of autism could account for the heterogeneity in the PMS group, we created a “proxy diagnosis” by using the scores from the ADOS and ADI-R. Participants who scored above cut off for autism or autism spectrum on the ADOS and above cut off in all three domains of the ADI-R were classified as having autism. We subsequently studied the differences in social and non-social orienting scores for those PMS individuals classified as having autism versus those who did not. We found no significant mean group differences in the scores between these two groups. Similarly, given the almost even proportion of males and females in the PMS, but not in the i-autism group, we studied differences between sexes in the PMS group to explore whether sex could be accounting for differences in performance. Again, results showed no significant mean group differences between both sexes in either task condition. Given these results, we decided to keep all PMS individuals together (irrespective of diagnosis or sex) to ensure a sample sufficiently large for analyses. Detailed analyses can be found in Appendix 2.

### Multiple linear regressions with autismrelated measures

To understand the association of task performance and clinical profile, orienting scores were examined in relationship to age (both chronological and mental), level of autism features (as measured by the ADOS-2 CSS), and level of social adaptive functioning (as measured by the Vineland-II). Regression coefficients and model fit statistics are presented in Table 2. These models explained 33% and 34% of the variance in social and non-social orienting in the PMS group and 39% in the i-autism group, only for non-social orienting. Mental ages combined with social adaptive ability were significant for the responses to both stimuli in the TD control group. While severity of autistic features predicted the number of orienting responses in both models for the PMS group, in autistic individuals, verbal and non-verbal mental ages predicted most variance in non-social orienting behavior.

**Table 2:** Multiple linear regressions models for both types of orienting scores with verbal and non-verbal mental ages, Vineland Socialization sub-domain scores, and ADOS-2 calibrated severity scores, by group

	**PMS**									
	**Social**					**Non-Social**				
	***B***	***SE B***	***β***	***R*** ^***2***^_***adj***_	***Model fit***	***B***	***SE B***	***β***	***R*** ^***2***^_***adj***_	***Model fit***
Constant	3.613	1.001				2.640	1.026			
NVMA	-0.007	0.019	-0.124			-0.005	0.019	-0.075		
VMA	0.034	0.018	0.592			0.028	0.018	0.462		
VABS Soc	-0.011	0.011	-0.134			0.005	0.011	0.063		
ADOS CSS	-0.201	0.080	-0.315*	.327	*F*_(4,54)_ = 6.569, *p* < .001***	- .0206	0.082	-0.313*	.340	*F*_(4,54)_ = 6.97, *p* < .001***
	**i-autism**									
	**Social**					**Non-Social**				
	***B***	***SE B***	***β***	***R*** ^***2***^_***adj***_	***Model fit***	***B***	***SE B***	***β***	***R*** ^***2***^_***adj***_	***Model fit***
Constant	0.866	1.082				0.672	0.996			
NVMA	-0.009	0.008	- .3608			-0.022	0.007	-0.814**		
VMA	0.025	0.013	0.559			0.054	0.012	1.143***		
VABS Soc	0.013	0.011	0.198			-0.003	0.010	-0.043		
ADOS CSS	0.007	0.098	0.012	.167	*F*_(4,32)_ = 1.600, *p* = .198	0.028	0.090	0.043	.394	*F*_(4,32)_ = 5.214, *p* = .002**
	**TD**									
	**Social**					**Non-Social**				
	***B***	***SE B***	***β***	***R*** ^***2***^_***adj***_	***Model fit***	***B***	***SE B***	***β***	***R*** ^***2***^_***adj***_	***Model fit***
Constant	2.064	2.126				2.253	1.515			
NVMA	-0.013	0.036	-0.170			-0.030	0.026	-0.649		
VMA	-0.008	0.027	-0.157			0.021	0.019	0.609		
VABS Soc	0.005	0.023	0.047	- .001	*F*_(3,23)_ = 1.483, *p* = .413	0.010	0.016	0.131	- .064	*F*_(3,23)_ = 0.480, *p* = .70

NVMA = Non-Verbal Mental Age; VMA = Verbal Mental Age; VABS = Vineland Adaptive Behavior Scale; Soc = Socialization; ADOS = Autism Diagnostic Observation Schedule; CSS = Calibrated Severity Score; B = coefficient B; SE = standard error; *β*: standardised regression coefficient; *R*^*2*^_*adj*_ = adjusted R squared; Residuals from regression models were approximately normally distributed and collinearity diagnostics suggested no multicollinearity between variables. *p < .05, **p < .01, ***p < .0125 (significant after Bonferroni correction; *p* = .05/4).

## Discussion

This study compared orienting responses to social and non-social stimuli between individuals with PMS, autistic children and neurotypical children, using a well-known semi-naturalistic social orienting paradigm [17]. Based on the social motivation hypothesis, we predicted that both clinical groups would react less often specifically to social but not non-social stimuli, as previously demonstrated in samples of idiopathic autistic children. We also tested the competing hypothesis that lower responses to either stimulus type may be related to intellectual disability. Findings showed that although both clinical groups responded less often to social stimuli than the neurotypical group (and did not significantly differ from each other), within-group comparisons by stimulus type revealed that the i-autism group responded relatively more often to social than non-social stimuli. The PMS group showed a different pattern, displaying on average lower rates of social and non-social orienting, in line with more domain-general attentional difficulties. Hence, our results did not replicate previous findings [17, 45] of specific difficulties with social orienting in autistic children and they did not find this pattern in the PMS group.

There are several factors that may have contributed to the unexpected finding in the i-autism group. Autistic participants in this study were generally older and represented a broader range of ages than some previous studies of social orienting to match the age range in the PMS group. Age and verbal mental age have been shown to play an important role in the acquisition of other aspects of social cognition in autism, such as a theory of mind [46, 47]. Perhaps there are true difficulties in social orienting in the early years, and that (some) autistic children learn with age, or receive training through intervention, to orient to relevant stimuli of social content, and to disregard background environmental noises (e.g., car horn, etc.) [48]. Unfortunately, we did not collect information on earlier development or possible interventions, and so could not test this hypothesis. These possibilities are, however, consistent with more recent studies of social attention in i-autism using eye-tracking tasks that also did not find universal difficulties in attending to social stimuli [49], including in older children [50].

In contrast, group level findings in the PMS group suggest similar difficulties in responding to both social and non-social stimuli. This finding is in line with previous results from a passive listening task in a small sample of children with PMS showing no differences in brain responses to social vs. non-social sounds while undergoing an MRI [51]. Given that the PMS group was, on average, mentally younger than the autistic participants, it is possible that discrimination between relevant and irrelevant stimuli has not yet been developed and could account for this lack of differences. Similarly, given that all our participants presented with intellectual disability, this could be the reflection of their overall developmental delay, and the impact of a delayed processing speed and/or mental capacity.

The present findings also highlight considerable individual differences in social and non-social attention in both clinical groups. Studies often focus on mean group differences but neglect the rich information that individual patterns provide. This is particularly notable in studies with rare genetic conditions, which often rely on small sample sizes. However, with increasing recognition of heterogeneity of autism, some studies have begun to examine individual differences in social attention among autistic individuals [49] and their relevance to other clinical features [52]. Indeed, at both our clinical sites we found ample variability of orienting responses not only among the i-autistic participants but also among the participants with PMS. While some individuals oriented to all stimuli regardless of their nature, others oriented to almost none. Yet other individuals showed a preference for either social or non-social stimuli.

To explore the relationship between orienting responses and clinical heterogeneity, we examined the role of mental age, autistic features and social adaptive behavior in the responses to social and non-social bids. This revealed differential relationships in the three groups. Contrary to the hypothesis that social orienting impacts social-communication, in the i-autism group, autistic features did not explain variance in the number of social orienting responses. Instead, higher verbal mental age and lower non-verbal mental age predicted more frequent orienting to non-social stimuli. It appears that as autistic children’s verbal abilities increase, so too does their ability to orient to non-social auditory stimuli, which could help increasing flexibility. In the neurotypical children group, social orienting was neither related to verbal or non-verbal mental age or level of social adaptive behavior. These findings also indicate that within the current age/mental age brackets, social orienting behavior alone could not predict clinically relevant levels of autistic features and/or adaptive behavior in autistic and neurotypical children.

By contrast, in the PMS group responses to both types of stimuli were strongly related to level of autistic features. This finding suggests that orienting behavior (regardless of stimulus type) may be of clinical relevance in PMS. If this finding replicates in other samples and shows good test-retest reliability, spontaneous orienting behavior may be explored as an objective measure to assess the efficacy of therapeutic interventions targeting autistic features in PMS. The social orienting paradigm is brief, easy to administer and score, and, unlike other measures of social attention (i.e., eye-tracking), does not rely on expensive equipment or require participants to sit still and attend for long periods of time. This may make social orienting a particularly useful tool among this population.

This study had several key strengths. To date, relatively little research has compared similarities or differences in social cognitive development between individuals with PMS and autistic individuals. This is due to the fact that most social cognitive paradigms are unsuitable for children with severe or profound intellectual disability. The social orienting task is well suited for people with severe or profound intellectual disability, and arguably more naturalistic than even eye-tracking tasks (which require participants to look at a screen); therefore results may be more representative of real-life responses. Furthermore, given the rarity of PMS, it is noteworthy that we have combined a relatively large sample of participants with PMS across two sites to examine behavioral differences in a labbased context. This enabled us to to begin investigating individual differences among people with PMS.

Some limitations are also worth noting. First, there were slight differences in administration between sites in the location pattern and amount of stimulus delivery. Statistical measures were nevertheless taken to minimize the impact of such differences. Another limitation is the difference in mental age between all three groups. Second, although the i-autism group had on average higher IQ than the PMS group, considerable efforts were made to recruit autistic children with moderate to severe ID. This represents a subgroup of autistic people that is often overlooked in clinical research. Third, non-parametric post-hoc comparisons were not corrected for multiple comparisons, which highlights the need for independent replication. Finally, as is typical in studies with autistic individuals, the male to female ratio was significantly more uneven than in the other two groups. To mitigate this problem, we tested for possible orienting differences in the other clinical group (i.e., PMS) and found no difference between sexes, concluding that sex, on this occasion, was not a relevant variable for performance in the PMS group.

## Conclusions

This study compared social and non-social orienting patterns between individuals with PMS, idiopathic autistic children and neurotypical children using a semi-naturalistic auditory orienting task. Both clinical groups responded less often to social stimuli compared to the neurotypical children. However, whereas the social motivation hypothesis of autism predicts selectively diminished attention to social stimuli, the PMS group oriented on average to both stimulus types at the same rate, while the i-autism and typically developing groups responded on average significantly more often to social than non-social stimuli. In addition, we also observed notable individual differences in orienting pattern in both clinical groups. This is particularly significant for the PMS group as most previous studies of PMS (and rare genetic syndromes) assumed greater homogeneity relative to idiopathic autism and rarely studied individual variability. Future studies will need to further examine the relationship between neurocognitive profile and clinical features in PMS and may explore the potential of orienting behaviours as outcome measure in clinical trials.

## Electronic supplementary material

Below is the link to the electronic supplementary material.

Supplementary Material 1

## Data Availability

The datasets used and/or analysed during the current study are available from the corresponding author on reasonable request.

## Footnotes

1 Henceforth referred to as autism in accordance with the preferred language of the autistic community.

## Abbreviations

ABC: Adaptive Behavior Composite
ADI-R: Autism Diagnostic Interview – Revised
ADOS-2: Autism Diagnostic Observation Schedule 2nd Edition
ASD: Autism Spectrum Disorder
CSS: Calibrated Severity Score
DMS: Diagnostic and Statistical Manual for Mental Disorders
i-autism: idiopathic autism
ICD: International Classification of Diseases
MRI: Magnetic Resonance Imaging
MSEL: Mullen Scales of Early Learning
NVIQ: Non-Verbal Intelligent Quotient
NVMA: NonVerbal Mental Age
PMS: Phelan-McDermid Syndrome
RRB: Repetitive and Restrictive Behaviors
SA: Social Affect
TD: Typically Developing
Vineland-II: Vineland Adaptive Behavior Scales 2nd Edition
VIQ: Verbal Intelligence Quotient
VMA: Verbal Mental Age
