# Supplementary material for: Investigating social orienting in children with Phelan-McDermid syndrome and ‘idiopathic’ autism
Source: J Neurodev Disord. 2024 Nov 19;16:64. doi: 10.1186/s11689-024-09564-7 (PMC11575217; doi:10.1186/s11689-024-09564-7)
Supplement: Supplementary file 1 — Supplementary Material 1 [file 11689_2024_9564_MOESM1_ESM.docx]

# Appendices

## **Appendix 1. Counterbalancing analyses**

### Counterbalancing analyses: back vs front presentation for the UK sample

|  | Front | Behind | *p* |
| --- | --- | --- | --- |
| Whole sample |  |  |  |
| Social | 2.59(1.21) | 2.08(1.29) | .015 |
| Non‑social | 1.75(1.37) | 1.49(1.42) | <.001 |
| All | 4.34(2.12) | 3.53(2.31) | <.001 |
| PMS |  |  |  |
| Social | 2.00(1.54) | 1.37(1.31) | .041 |
| Non‑social | 1.63(1.56) | 1.18(1.51) | <.001 |
| All | 3.62(2.78) | 2.37(2.22) | <.001 |
| i‑autism |  |  |  |
| Social | 2.68(1.09) | 1.72(1.20) | .025 |
| Non‑social | 1.50(1.30) | 1.12(1.07) | <.001 |
| All | 4.19(1.91) | 2.85(1.74) | <.001 |
| TD |  |  |  |
| Social | 2.88(0.97) | 2.78(1.01) | .56 |
| Non‑social | 2.03(1.28) | 1.98(1.52) | .92 |
| All | 4.92(1.68) | 4.76(2.25) | .73 |

### Counterbalancing analyses: 1st vs 2nd presentation for the UK sample

|  | 1^st^ presentation | 2^nd^ presentation | *p* |
| --- | --- | --- | --- |
| Whole sample |  |  |  |
| Social | 2.18(1.31) | 2.50(1.23) | .061 |
| Non‑social | 1.55(1.40) | 1.65(1.37) | .549 |
| All | 3.70(2.26) | 4.15(2.22) | .133 |
| PMS |  |  |  |
| Social | 1.37(1.36) | 2.00(1.50) | .094 |
| Non‑social | 1.41(1.62) | 1.39(1.48) | .952 |
| All | 2.65(2.42) | 3.39(2.71) | .223 |
| i‑autism |  |  |  |
| Social | 1.95(1.26) | 2.50(1.18) | .081 |
| Non‑social | 1.15(0.94) | 1.33(1.28) | .580 |
| All | 3.11(1.85) | 3.83(1.91) | .189 |
| TD |  |  |  |
| Social | 2.85(0.99) | 2.81(1.00) | .881 |
| Non‑social | 1.96(1.50) | 2.06(1.31) | .818 |
| All | 4.81(2.02) | 4.87(1.94) | .909 |

## **Appendix 2. Group differences in the PMS group by autism diagnosis and sex (mean and SD)**

|  |  | *n* | *Social* | *p* | *Non-Social* | *p* |
| --- | --- | --- | --- | --- | --- | --- |
| Autism | *yes* | 40 | 2.24 (1.26) | .78 | 1.92 (1.42) | .35 |
|  | *no* | 22 | 2.11 (1.37) |  | 2.27 (1.42) |  |
| Sex | male | 33 | 2.27 (1.23) | .63 | 2.15 (1.38) | .73 |
|  | female | 30 | 2.10 (1.35) |  | 2.01 (1.32) |  |
